# The drivers of non-adherence to albuminuria testing guidelines and the clinical and economic impact of not identifying chronic kidney disease 

**DOI:** 10.5414/CN111106

**Published:** 2023-08-30

**Authors:** Franziska Groehl, Antonio Garreta-Rufas, Kimberley Meredith, James Harris, Peter Rossing, F. D. Richard Hobbs, Christoph Wanner

**Affiliations:** 1 Bayer AG, Berlin, Germany,; 2 Wickenstones Ltd, Carlow, Ireland,; 3 Steno Diabetes Center, Copenhagen,; 4 Oxford Primary Care, Radcliffe Primary Care Building, Radcliffe Observatory Quarter, University of Oxford, Oxford, UK, and; 5 Center of Internal Medicine, University Hospital, Würzburg, Germany

**Keywords:** chronic kidney disease, urine albumin-to-creatinine ratio, albuminuria, glomerular filtration rate, diagnosis

## Abstract

Background: Regular monitoring is required to ensure that patients who have, or are at risk of, chronic kidney disease (CKD) receive appropriate management. Guidelines recommend regular testing of estimated glomerular filtration rate (GFR) and albuminuria. However, evidence suggests that albuminuria testing rates, specifically urine albumin-to-creatinine ratio (UACR), are suboptimal. Aim: To assess published evidence relating to the drivers of non-adherence to albuminuria testing guidelines and the impact of not identifying CKD across the course of progression. Materials and methods: A systematic review of five bibliographic databases was conducted, supplemented by hand searches of relevant conference abstracts. Results: One study was identified that reported drivers of non-adherence to albuminuria testing guidelines. The largest barrier was the perception that testing does not impact patient management. Thirteen studies were identified that evaluated the impact of not identifying CKD patients. All included studies analyzed the effect of not identifying worsening CKD severity leading to late referral (LR). 12/13 studies reported only on clinical impact, and 1/13 reported on clinical and economic impact. LR led to higher costs and worse outcomes than early referral, including higher rates of mortality and worsened kidney replacement therapy preparation. Conclusion: This systematic review demonstrates a gap in evidence exploring the drivers of non-adherence to albuminuria testing guidelines and the impact of not identifying patients in the early stages of CKD. Guideline-recommended testing allows timely identification, referral, and treatment for patients with, or at risk of, CKD, providing the best chance of avoiding the worsened outcomes identified in this review.

## Introduction 

Chronic kidney disease (CKD) poses diagnostic challenges due to asymptomatic progression in its early stages, with symptoms only presenting once substantial kidney function has been lost [1]. As CKD progresses to more advanced stages, such as end-stage kidney disease (ESKD), there is a strong association with worsening clinical outcomes, economic burden, and healthcare resource utilization [2]. In more advanced stages, patient management options may also be limited to co-morbidity management, dialysis, and/or kidney transplantation [3]. 

When detected early, CKD progression is an increasingly manageable disease, due to the availability of established and new treatment options [4, 5, 6]. To allow for timely initiation of these treatments, comprehensive and regular testing of at-risk populations is recommended by global guidelines [3, 6, 7]. This includes estimated glomerular filtration rate (GFR) testing, an established method for monitoring kidney function, and testing for albuminuria, an indicator of kidney damage and cardiovascular (CV) event risk. Dual testing is crucial, as CKD-related kidney damage can be present even when estimated GFR indicates normal kidney function, as evidenced by high levels of albuminuria [3]. 

Diabetes [6], CV [7], and kidney disease guidelines [3] recommend simultaneous dual testing (with the quantitative urine albumin-to-creatinine ratio (UACR) test being preferred over less sensitive urine albuminuria dipstick testing). Despite GFR testing guidelines being well adhered to, real-world evidence suggests that physicians are not consistently adhering to albuminuria testing guidelines [8]. Low rates of UACR testing have been reported in countries such as France (29%) [9] and the US (21%) [10]. As yet, it is unclear why there is underuse of UACR testing. 

Optimized identification of CKD as per guidelines, using both methods of testing, is likely to have benefits to how care is delivered as well as to patient outcomes [1]. However, from our knowledge, no studies have systematically examined the impact of not identifying CKD patients. 

The present study sought to understand, via a systematic review, reasons for non-adherence to albuminuria testing guidelines, including UACR testing, in CKD and at-risk populations. In addition, we evaluated the clinical and economic impact of not identifying CKD across the course of a patient’s disease progression – this includes failed/late/missed/no diagnosis, and lack of identification of worsening CKD severity leading to late referral for kidney replacement therapy (KRT). 

## Materials and methods 

### Research questions 

This systematic review asked the following research questions via a single search strategy (see ‘Appendix’ for search strategy):

- What are the drivers of non-adherence to albuminuria testing guidelines in diagnosing and tracking CKD progression?
- What is the clinical/humanistic and economic impact of not identifying CKD over the course of progressive kidney function loss?

### Research methods 

The systematic review was conducted in accordance with the Centre for Reviews and Dissemination handbook and reported in accordance with the Preferred Reporting Items for Systematic Reviews and Meta-analyses (PRISMA) recommendations [11]. The protocol was pre-registered on the PROSPERO international register (CRD42021275223). 

### Literature search 

This review used search terms relating to CKD, albuminuria, and non-identification. Five databases (see Supplemental material) were searched from the date of database inception to August 24, 2021. In addition, forward citation searching using Science Citation Index Expanded was used. Abstracts from 8 conferences (see Supplemental material), limited to 2020 – 2021, were hand-searched by a single researcher (KM). In addition, backwards citation searching was undertaken on any systematic reviews that met the inclusion criteria at the title and abstract (ti/ab) stage. While this document will use recent nomenclature definitions from the Kidney Disease Improving Global Outcomes (KDIGO) consensus conference [12], search strings account for the historic variability in language. 

### Screening 

Two researchers independently screened ti/ab (KM and IMG) and full texts (KM and RT) of records for both research questions simultaneously. Disagreements over study eligibility were resolved by a third researcher (JH). As documented in PROSPERO, during the ti/ab screening process, studies were tagged as either CKD-only, or type 2 diabetes (T2D) with CKD patient populations, allowing the included population to be broadened to CKD patients if a limited number of T2D-specific papers were captured. 

### Inclusion and exclusion criteria 

Each research question had its own inclusion and exclusion criteria (Table 1). Records were screened against both criteria, however, records only had to meet one of the research question’s criteria to be included. 

### Data extraction and quality assessment 

Data, which included record information, study design, and outcomes were extracted by one reviewer (KM), and independently checked by a second reviewer (RT) for accuracy and completeness. Study quality was assessed by two researchers independently using the National Heart, Lung, and Blood Institute quality assessment tool for observational, cohort, or cross-sectional studies [13]. In addition, the Strengthening the Reporting of Observational Studies in Epidemiology checklist for cross-sectional studies was used for surveys [14]. 

## Results 

### Research question 1 – drivers of non-adherence to albuminuria testing guidelines 

From a total of 13,271 records screened, only 1 record met the inclusion criteria for research question 1 [15]. The study was a cross-sectional survey of active US primary care physicians on reasons for non-adherence to albuminuria testing guidelines in non-diabetic patients [15]. Physicians were asked to agree or disagree with a selection of barriers presented in two clinical settings – patients with hypertension and eGFR ≥ 60 mL/min/1.73m^2^, and patients with hypertension and eGFR < 60 mL/min/1.73m^2^. The perception was that albuminuria test results would not significantly impact management (37% and 24%, for eGFR ≥ 60 and eGFR < 60 mL/min/1.73m^2^, respectively). The following factors were also listed as barriers to albuminuria testing (largest to smallest): limited time or more urgent patient issues (25% and 20%), not recommended by guidelines (25% and 11%), cost (13% and 9%), and poor patient adherence (5% and 5%). 

### Research question 2 – the impact of not identifying chronic kidney disease patients 

**Figure 1.**
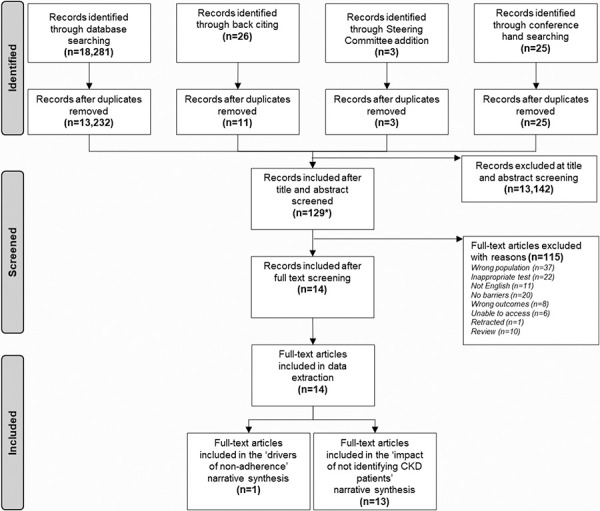
PRISMA flow diagram of included publications. *Only 11 of the 129 records captured type 2 diabetes patients with chronic kidney disease. Therefore, the patient population was broadened to chronic kidney disease patients. CKD = chronic kidney disease.

Based on a pre-defined criterion (see “Research methods”), the population for research question 2 was broadened from T2D CKD patients to CKD patients at the ti/ab screening stage (ahead of full-text screening) because only 11 records specifically captured T2D patients with CKD [16, 17, 18, 19, 20, 21, 22, 23, 24, 25, 26]. Therefore, after broadening the criteria, from a total of 13,271 records screened, 13 records were included for research question 2 (Figure 1). 

All 13 included records reported on different studies. All included studies (13/13) reported clinical and/or humanistic outcomes; 1 of these studies (1/13) also reported economic outcomes. All studies (13/13) included GFR testing; no studies (0/13) used UACR testing for diagnostic purposes. Henceforth, the term GFR includes the terms estimated GFR and residual GFR used by some included studies. 

Of the 13 included studies, 5 were conducted in Europe [27, 28, 29, 30, 31], 5 in Asia [32, 33, 34, 35, 36], 2 in the USA [37, 38], and 1 in South America [39]. Study cohort sizes ranged from 75 to 2,195 participants. One included study analyzed only children (< 18 years old) [27]; the remaining studies (12/13) evaluated adults. 

All studies (13/13) were focused on understanding the impact of not identifying progression through analyzing the impact of early vs. late referral time to a nephrologist; no studies (0/13) were identified that examined the impact of non-diagnosis. Studies classified late referral (LR) either by the time-period between specialist nephrology referral and start of KRT (10/13 studies) [27, 28, 29, 31, 32, 33, 34, 35, 36, 37] or the GFR value at referral (3/13 studies) [30, 38, 39]. The definition of late referral by time varied from < 1 month to < 1 year between seeing a nephrologist and commencing KRT. GFR value for late referral was either defined as < 15 mL/min/1.73m^2^ or < 20 mL/min/1.73m^2^. 

One study segmented an early referral (ER) sub-population by planned or unplanned dialysis (Table 2) [28]. 

### Impact of late referral on glomerular filtration rate 

All included studies recorded GFR testing at time of referral (baseline) and/or time of KRT commencement; none reported albuminuria testing. 

**Figure 2.**
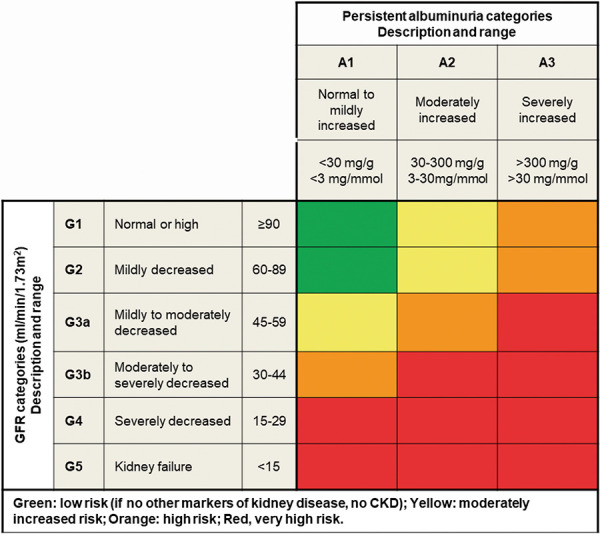
Prognosis of chronic kidney disease by glomerular filtration rate and albuminuria categories: KDIGO 2012 [3]. CKD = chronic kidney disease; GFR = glomerular filtration rate.

GFR at time of referral was measured in 8 studies, of which 7 reported lower GFR in LR than ER [27, 29, 31, 32, 33, 34, 36], demonstrating an association between late referral and poorer kidney function. Five comparisons were statistically significant (p < 0.05), ranging from 4.4 – 14.9 mL/min/1.73m^2^ for LR and 5.1 – 45.6 mL/min/1.73m^2^ for ER [27, 29, 32, 33, 34]. LR cohorts in all studies reported a mean GFR within the CKD G5 region (Figure 2), regardless of the time between referral and KRT initiation. 

Four studies recorded GFR at the time of KRT [27, 28, 32, 34]. Irrespective of referral group (late or early), all GFR mean values were in the G5 region (Figure 2). Two studies recorded lower GFR in LR than ER at the time of KRT, with only 1 of these studies showing statistical significance (7.9 vs. 10.2 mL/min/1.73m^2^; p = 0.02) [27]. 

### Impact of late referral on mortality and cardiovascular disease 

All studies that reported mortality (across all LR definitions, follow-up lengths, and types of KRT) (9/13) reported a higher mortality rate amongst patients experiencing an LR compared with an ER for CKD [29, 30, 31, 32, 33, 35, 36, 37, 38]. Within this subgroup of 9 studies, all those that performed unadjusted hazard models (7/9) reported an increased risk of mortality with LR [29, 30, 31, 32, 35, 36, 37] compared with ER; 5 of these reported a statistically significant risk, ranging from hazard ratio (HR), 2.1; 95% confidence intervals (CI), 1.6 – 2.9 to HR, 2.68; 95% CI, 1.20 – 5.98 [29, 31, 32, 36, 37]. 

In studies where models were adjusted for relevant factors (4/9), LR retained its association with greater mortality risk in all but 1 study (HRadjusted, 0.52; 95% CI, 0.22 – 1.22)) [36], where adjustments for baseline serum albumin concentration and residual GFR were made. One study adjusted for estimated GFR, along with 10 other laboratory measurements where LR was associated with mortality (HRadj, 2.38 (95% CI: 1.27 – 4.45)) [32]; however, in this study, GFR did not significantly differ between LR and ER cohorts. All remaining studies that had adjusted for factors reported an increased risk of mortality with LR [29, 31, 37]. 

The relationship between GFR and all-cause mortality was not consistent across all studies. Within 3 studies [31, 32, 36], lower GFR – indicating worse renal function – either measured at the time of referral or dialysis, was significantly associated with increased mortality. However, 2 studies reported an increased risk of mortality with higher GFR when recorded at dialysis [35, 37]. In 1 study, this was explained to be due to patient characteristics – those with a higher GFR being older, occupationally less active, having more comorbidities, and poorer nutritional status than those with lower GFR [35]. In the remaining study, those who were referred late had a higher average GFR than those referred early, 9.1 vs. 8.7 mL/min/1.73m^2^, respectively [37]. 

LR was associated with a five-fold increased risk of CV disease when LR was defined as < 3 months (HR univariate, 5.43 (95% CI: 1.46 – 20.21), p = 0.01) [36] and < 1-year (HR multivariate, 4.99 (95% CI: 1.48 – 16.82), p = 0.009) [32]. 

### Impact of late referral on mortality in patients with diabetes 

Diabetes mellitus sub-groups were analyzed in 2 studies, neither of which specified the proportion of type 1 diabetic vs. T2D patients [29, 32]. In a univariate analysis of one such sub-group, LR (defined as < 1 year) was associated with an increased risk of mortality (HR, 2.42, (95% CI: 1.26 – 4.64); p = 0.008) and CV death (HR, 26.71 (95% CI: 1.49 – 478.99, p = 0.03) [32). In the other study, an adjusted model evaluating patients with diabetes disclosed a similar trend between LR (defined as < 3 months) and mortality [29]. However, through a synergy index calculation, diabetes did not have an additional worsening effect, inferring that timely referral is important in future dialysis patients, irrespective of diabetic status. 

### Impact of late referral on hospitalization 

Across 3 studies that reported on hospitalization, none reported statistically significant trends between LR and hospitalization across various LR definitions [35, 36, 37]. In 1 study, GFR was an independent predictor for all-cause hospitalization (HR, 1.075 (95% CI: 1.011 – 1.42); p = 0.02) [35]. 

### Impact of late referral on dialysis 

**Emergent dialysis**

One study reported that the rate of emergent dialysis was lower in ER (0%) than in LR (53%) (defined as < 3 months) [33]. Moreover, emergency hemodialysis (HD) was associated with an increased rate of mortality when LR was defined as < 12 months (HR, 1.63 (95% CI 1.00 – 2.67)) [32]. 

**Dialysis modality**

Analysis of dialysis modality was recorded in 4 studies [27, 28, 29, 38]. In a study of children, ER patients were significantly more likely than LR (defined as < 3 months) patients to undergo the preferred guideline modality of kidney transplantation (40% vs. 11%; p = 0.007) and < 12 months (40% vs. 21%; p = 0.04) [27]. Despite this, GFR did not correlate with pre-emptive kidney transplantation (odds ratio, 0.99 (95% CI, 0.98 – 1.01; p = 0.4)). 

One study (LR defined as < 1 month) demonstrated a higher rate of peritoneal dialysis (PD) in ER patients compared with LR patients (29% vs. 11%; p = 0.002) [28]. When the LR definition was increased from < 1 month to < 90 days, the significant difference appeared to be mitigated (32% vs. 20%; p = 0.06). A non-diabetic CKD population that defined LR by a specific GFR (< 15 mL/min) reported a non-significant higher rate of receiving PD as first KRT in ER patients compared with LR patients (p = 0.4) [38]. In an additional study, where rates of HD were analyzed and LR was defined < 3 months, LR patients started more frequently on this modality compared with ER patients; no data nor p-values were provided [29]. 

**Vascular access**

The timing and type of vascular access differed between referral groups (late vs. early), which appeared to impact clinical outcomes, particularly mortality. 

In studies that analyzed vascular access in patients undergoing HD (n = 4), ER was associated with higher rates of permanent vascular access (PVA) compared with LR across various LR definitions [28, 31, 34, 38]. Within the ER cohort of Caskey et al., planned HD patients were more likely to have PVA compared with unplanned patients (p < 0.001) [28]. Additionally, Selim et al. [31] demonstrated that temporary vascular access vs. arteriovenous fistula (AVF) – a PVA method – was associated with an increased risk of all-cause mortality within 5 years after HD initiation (HR, 1.68 (95% CI: 1.02 – 2.75); p = 0.04)). 

One study analyzing patients undergoing PD demonstrated that ER was associated with a higher likelihood of planned implantation of catheters than unplanned (p < 0.001), where unplanned catheter implantation was associated with an increased risk of all-cause hospitalization (HR, 1.694 (95% CI: 1.111 – 2.583); p = 0.01), but not mortality (HR, 1.643 (95% CI: 0.689 – 3.915)) [35]. 

**Impact of late referral on health-related quality of life**

Only 1 study analyzed the effect of LR (< 1 month) on health-related quality of life (QoL), where the visual analog scale (VAS) and Short Form 36 (SF-36) instruments were undertaken 8 weeks after dialysis initiation [28]. VAS scores were significantly higher (i.e., a better QoL) in ER patients compared with LR patients (58.4 vs. 50.4; p = 0.005), although this difference was not significant on exclusion of a patient subgroup from Estonia for whom SF-36 data were unavailable (p = 0.09). When measured by SF-36, ER had no independent effect on QoL, on either the Physical Component Summary (PCS) score (p = 0.4) or the Mental Component Summary (MCS) score (p = 1.0). By comparison, when a sub-group analysis of planned vs. unplanned dialysis within the ER group was undertaken, patients undergoing planned dialysis had a significantly better mean QoL (VAS) compared with unplanned dialysis (p = 0.03) and significantly higher MCS scores (p = 0.003), but insignificant PCS scores [28]. 

The percentage of good nutrition was measured using the subjective global assessment in 2 studies, with better nutritional status observed in ER vs. LR (32.7% vs. 0%) [39] and (71% vs. 63.2%) [29]. 

**Impact of late referral on healthcare costs**

The 1 study capturing costs defined LR as < 1 year [34]. The total medical costs during the first 12 months after dialysis initiation did not differ between ER and LR (p = 0.8). By comparison, costs during the first month after dialysis were statistically higher in LR patients compared with ER patients (3,438 vs. 3,029 USD; p = 0.03), which was driven partly by dialysis type (HD) (p < 0.001). More patients in ER vs. LR started HD with a native AVF (30.7 vs. 20.1%; p = 0.003) and without the insertion of a temporary vascular central venous catheter (40.2 vs. 49.0%; p = 0.03). Both variables contributed towards the lower costs seen in ER patients. In the year before dialysis, ER was associated with lower medical costs compared with LR (p < 0.001). The relationship between medical costs and laboratory parameters was not investigated. 

## Discussion 

### Reasons for non-adherence to albuminuria guidelines 

In the single study [15] identified in this systematic review addressing reasons for non-adherence to albuminuria guidelines, the most prominent reason stated was that test results would not significantly impact non-diabetic patient management, i.e., even if the test was done, results would not have influenced choice of the limited treatment options at the time (2014). However, with additional treatment options becoming increasingly available that are able to slow CKD progression, this rationale will become harder to justify for decision makers. As the patient population of interest in the above study did not have T2D – a common risk factor for CKD – there is a high likelihood that this drove the understanding that testing for albuminuria is not recommended by guidelines for this patient population. 

The lack of evidence identified through the systematic review prompted some of the authors to conduct a short global survey of healthcare practitioners to explore reasons further. This reported that the most prominent reason (27% of all reasons provided) was lack of awareness of guidelines/UACR testing [40], demonstrating a need for increased education aimed at healthcare practitioners. 

### Paucity of evidence examining the impact of not identifying chronic kidney disease patients 

Research question 2 within the review set out to explore the impact of not identifying CKD patients, a possible scenario when CKD testing guidelines are not adhered to. 

No included studies analyzed the impact of not identifying patients in the early stages of CKD. This may be due to the difficulty in identifying these patients due to the asymptomatic nature of CKD in the early stages. In addition, the search strategy included terms for diagnosing, meaning that these types of papers should have been captured by this literature review, again evidencing for a lack of literature on this topic. 

Instead, all 13 studies analyzed the impact of not identifying progression of CKD, as these studies were focused on late vs. early referral to a nephrologist. As only early vs. late referral cohorts were identified, with GFR (which measures kidney function) being the only CKD diagnostic test captured, it could be inferred that kidney damage measured by testing for albuminuria test is a hidden confounder of the outcomes evaluated. Further investigation into the outcomes associated with dual testing in a patient population other than early vs. late referral is required to observe the complete benefits of early CKD identification. 

QoL and economic impact of not identifying CKD patients were underexplored outcomes, as were outcomes relating to diabetic patients. Table 3 summarizes the questions that require answering to fill in the numerous evidence gaps identified by this review. 

### The clinical impact of late referral 

All 13 studies consistently pointed to worse clinical and economic outcomes in CKD patients who were referred late. 

Timely referral is a key component of optimal CKD care; therefore, it could be assumed that patients who were referred late received suboptimal care across the course of their progression, which includes late initiation of therapy. Timely initiation is critical to managing CKD patients as it slows progression, reduces secondary processes that contribute to ongoing nephron loss, and can manage patients’ high blood pressure [41], a potential contributor to CV events. However, for patients to be initiated on timely and accurate therapy, patients must receive regular eGFR testing and testing for albuminuria to assess their true CKD severity. 

The clinical urgency to initiate KRT is likely to be high in those referred late, leading to limited preparation time – a potential driver to the worse clinical outcomes identified by this review. During the preparation stages ahead of dialysis, many guidelines recommend PVA over temporary vascular access. However, PVA requires timely assessment of the suitability for, and creation of, pre-emptive access [42]. Therefore, the higher rates of PVA in ER cohorts where time for preparation ahead of dialysis is longer than LR, are not unexpected. This preferred method improves mortality rates, possibly through reducing systemic infection rates [43]. LR also resulted in an increased likelihood of patients receiving unplanned catheter implantation over planned implantation, with unplanned being associated with increased rates of hospitalizations [35]. This further supports the concept that LR leads to a lack of preparation time ahead of KRT, resulting in worse clinical outcomes for patients. 

Amongst patients referred early to a nephrologist, those undergoing planned dialysis had a better QoL than those who underwent unplanned dialysis [28]. Despite being referred early, the importance of opportune preparation ahead of initiating ESKD management is again highlighted. 

The clinical impact of LR (increased risk of all-cause mortality, CV mortality, and the likelihood of suboptimal preparation for the chosen dialysis modality) has been similarly observed in a meta-analysis solely focused on LR [44]. 

### The economic impact of late referral 

As only 1 study analyzed the economic impact of not identifying CKD patients, there is a need for further research in this area [34]. Nonetheless, that study demonstrated that patients referred late to nephrology services had increased healthcare costs. It appears that worse clinical outcomes, including higher mortality rates, and poor preparation, including frequent use of temporary vascular access, drove these higher costs. If superior clinical care and more timely preparation – in the form of ER – are primarily provided to patients with CKD, it can be assumed that the economic burden associated with LR can be simultaneously reduced. As the unidentified global CKD population continues to grow due to rising numbers of T2D patients and continued lack of monitoring, there needs to be increased awareness around the cost savings that could be achieved with identifying and referring patients in a timely manner [45]. 

### Limitations of this review 

Findings from this review should be interpreted with consideration of several limitations. The search strategy was focused on capturing English-language papers only, and there was no exploration of a psychology-specific database that may have contained literature relevant to the research questions. In addition, the strategy was designed to capture studies that analyzed the impact of not identifying CKD patients; however, it did not include explicit terms to specifically capture phrases related to referral – the only topic identified for research question 2. Studies that only had mention of referral and not other terms related to identification and diagnosing may have been missed. 

For research question 2, the included studies were published between 2003 and 2015, despite searching for studies up to August 2021. Therefore, outcomes may not be as representative of current clinical practice as they could be, which indicates that additional primary research into this area is warranted. 

## Conclusion 

This review demonstrates that there is a lack of evidence exploring reasons for not undertaking testing for albuminuria in patients with, or at risk of, CKD, and the impact of not identifying patients in the early stages of CKD. Included studies demonstrated that late referral is associated with worse clinical outcomes and higher health care costs. Patients should be regularly monitored as per guidelines and receive regular GFR and testing for albuminuria to increase the chance of timely referral. 

## Funding 

This study was funded by Bayer, AG and conducted by Wickenstones Ltd. 

## Conflict of interest 

FG reports personal fees from Bayer AG Division Pharmaceuticals, during the conduct of the study; personal fees from Bayer AG Division Pharmaceuticals, outside the submitted work. 

GR reports personal fees from Bayer AG Division Pharmaceuticals, during the conduct of the study; personal fees from Bayer AG Division Pharmaceuticals, outside the submitted work. 

KM is employed by Wickenstones Ltd, a company that received consultancy fees from Bayer. 

JH is employed by Wickenstones Ltd, a company that received consultancy fees from Bayer. 

PR reports grants and other from Bayer, during the conduct of the study; grants and other from AstraZeneca, other from Astellas, other from Boehringer Ingelheim, other from Novo Nordisk, other from Gilead, other from Abbott, other from Merck, other from Sanofi, outside the submitted work. 

FDRH reports grants from National Institute for Health Research (NIHR) School for Primary Care Research, NIHR Applied Research Collaboration, the NIHR Oxford Biomedical Research Centre (BRC), and the NIHR Oxford Medtech and In-Vitro Diagnostics Co-operative (MIC), outside the submitted work; and FDRH occasionally consults or lectures, usually linked to an international medical society event, for global biotech companies, which include Amgen, Bayer, BI, BMS, Novartis, Novo Nordisk, and Pfizer in the past 5 years on his specialty expertise in cardiovascular disease and digital studies. 

CW reports personal fees from AstraZeneca, personal fees from Amgen, personal fees from Bayer, personal fees from Boehringer Ingelheim, personal fees from FMC, personal fees from MSD, personal fees from GSK, personal fees from GILEAD, personal fees from Vifor, outside the submitted work. 

**Table 1.** Broad inclusion and exclusion criteria for systematic review research questions.

Research question	Characteristic	Inclusion criteria	Exclusion criteria
What are the drivers of non-adherence to albuminuria testing guidelines in diagnosing and tracking CKD progression?	Population	Health care professionals	Any other stakeholders
	Intervention	Testing for albuminuria, type of test not specified	Any other test, including GFR testing
	Outcome	• Drivers of non-adherence to testing guidelines • Barriers to testing guidelines	Any other outcomes
	Study design	Any	No restrictions
	Language	English language	Non-English language
What is the impact of not identifying CKD over the course of progressive kidney function loss?	Population	Patients with CKD that have not been identified, including the following: • Missed/late diagnosis (when a diagnosis was only made at a more advanced stage) • Failed diagnosis (when a diagnosis has not been correctly identified) • Not diagnosed	• Patients with type 1 diabetes and CKD • Any other patient population
	Intervention	To capture patients that had not been identified before, studies needed to include a CKD diagnostic test, including any of the following: • Test for albuminuria plus GFR test • GFR test only • Test for albuminuria only	Any other diagnostic tests
	Outcome	• Any clinical outcomes • Any economic outcomes	No restrictions on outcomes
	Study design	Any	No restrictions
	Language	English language	Non-English language

CKD = chronic kidney disease; GFR = glomerular filtration rate.

**Table 2.** Included studies and their definitions of late referral, additional definitions, and kidney replacement therapy types.

Study	Late referral definition*	Additional definitions	Renal replacement type^†^
Boehm et al. 2010 [27]	Time: ≤ 3 months	N/A	Hemodialysis, peritoneal, and kidney transplant
Caskey et al. 2003 [28]	Time: < 1 month	Planned (previous serum creatinine > 300 μmol/L and non-urgent first dialysis) vs. unplanned dialysis	Hemodialysis and peritoneal
Chow et al. 2008 [36]	Time: < 3 months	N/A	Peritoneal
De Jager et al. 2011 [29]	Time: < 3 months	N/A	Hemodialysis and peritoneal
Guerra et al. 2014 [39]	GFR value at referral: < 15 mL/min/1.73m^2^	N/A	Unspecified dialysis
Kazmi et al. 2014 [37]	Time: < 4 months	N/A	Hemodialysis and peritoneal
Kim et al. 2013 [32]	Time: < 1 year	N/A	Hemodialysis and peritoneal
Kumar et al. 2012 [33]	Time: < 3 months	N/A	Unspecified dialysis
Lee et al. 2014 [34]	Time: < 1 year	N/A	Hemodialysis and peritoneal
Lhotta et al. 2003 [30]	GFR value at referral: < 20 mL/min/1.73m^2^	N/A	Unspecified dialysis
Navaneethan et al. 2007 [38]	GFR value at referral: < 15 mL/min	N/A	Hemodialysis and peritoneal
Selim et al. 2015 [31]	Time: < 1 year	Early start dialysis vs. late start dialysis (GFR value of ≥ 7.5 mL/min/1.73m^2^)	Hemodialysis
Shiao et al. 2008 [35]	Time: < 6 months	Early start vs. late start dialysis (GFR value of > 5 mL/min/1.73m^2^)	Peritoneal and hemodialysis

*Time between seeing nephrologist and commencing kidney replacement therapy; †The following terms: hemodialysis, peritoneal dialysis, dialysis (type unidentified in included studies), and kidney transplant, are collectively referred to as kidney replacement therapy in this study. GFR = glomerular filtration rate; N/A = not applicable.

**Table 3.** Proposed research questions.

Proposed research questions
What is the clinical and economic impact of identifying CKD patients (with type 2 diabetes) through late diagnosis/referral vs. early diagnosis/referral when both eGFR tests and albuminuria tests have been undertaken? This research question should have a specific focus on the quality of life and costs measured across CKD progression, including diagnosis, referral to a nephrologist, and requirement for KRT.
What is the real-world impact of early diagnosis and timely treatment regimens that can prevent CKD progression, compared with late diagnosis where timely treatment and management are not given?

CKD = chronic kidney disease; eGFR = estimated glomerular filtration rate; KRT = kidney replacement therapy.

## Supplemental material

Search terms, databases searched, and hand-searched conferences
