# Supplementary material for: The drivers of non-adherence to albuminuria testing guidelines and the clinical and economic impact of not identifying chronic kidney disease
Source: Clin Nephrol. 2023 Aug 30;100(4):145–56. doi: 10.5414/CN111106 (PMC10548545; doi:10.5414/CN111106)
Supplement: Search terms, databases searched, and hand-searched conferences [file clinnephrol-100-145-S01.pdf]

Appendix

Search terms

A search of the following bibliographic databases was undertaken using the following search strategy: terms for chronic kidney disease AND ([terms for albuminuria testing]) OR [terms for not identifying]

Databases searched

- MEDALL via Ovid
- Embase via Ovid
- The Cochrane Central Register of Controlled Trials via Wiley
- The Cochrane Database of Systematic Reviews via Wiley
- Cumulative Index to Nursing and Allied Health (CINAHL) via EBSCOhost
  Conference Proceedings Citation Index

Hand-searched conferences

- European Renal Association
- American Society of Nephrology
- American Diabetes Association
- European Association for the Study of Diabetes
- European Renal Best Practice
- European Society of Cardiology
- American Heart association
- American College of Cardiology
